# Potential role of senescent macrophages in radiation-induced pulmonary fibrosis

**DOI:** 10.1038/s41419-021-03811-8

**Published:** 2021-05-22

**Authors:** Lulu Su, Yinping Dong, Yueying Wang, Yuquan Wang, Bowen Guan, Yanhua Lu, Jing Wu, Xiaochun Wang, Deguan Li, Aimin Meng, Feiyue Fan

**Affiliations:** 1 Beijing Key Laboratory for Animal Models of Emerging and Remerging Infectious Diseases, Institute of Laboratory Animal Sciences, Chinese Academy of Medical Sciences, 100021 Beijing, China; 2 NHC Key Laboratory of Human Disease Comparative Medicine, Comparative Medicine Center, Peking Union Medical College, 100021 Beijing, China; 3 Institute of Radiation Medicine, Chinese Academy of Medical Sciences and Peking Union Medical College, Tianjin Key Laboratory of Radiation Medicine and Molecular Nuclear Medicine, 300192 Tianjin, China; 4 The Beijing Prevention and Treatment Hospital of Occupational Disease for Chemical Industry, Beijing Institute of Occupational Disease Prevention and Treatment, 100093 Beijing, China

**Keywords:** Senescence, Chemokines

## Abstract

Radiation-induced pulmonary fibrosis (RIPF) is a late toxicity of therapeutic radiation in clinic with poor prognosis and limited therapeutic options. Previous results have shown that senescent cells, such as fibroblast and type II airway epithelial cell, are strongly implicated in pathology of RIPF. However, the role of senescent macrophages in the development RIPF is still unknown. In this study, we report that ionizing radiation (IR) increase cellular senescence with higher expression of senescence-associated β-galactosidase (SA-β-Gal) and senescence-specific genes (p16, p21, Bcl-2, and Bcl-xl) in irradiated bone marrow-derived monocytes/macrophages (BMMs). Besides, there’s a significant increase in the expression of pro-fibrogenic factors (TGF-β1 and Arg-1), senescence-associated secretory phenotype (SASP) proinflammatory factors (Il-1α, Il-6, and Tnf-α), SASP chemokines (Ccl2, Cxcl10, and Ccl17), and SASP matrix metalloproteinases (Mmp2, Mmp9 and Mmp12) in BMMs exposed to 10 Gy IR. In addition, the percentages of SA-β-Gal^+^ senescent macrophages are significantly increased in the macrophages of murine irradiated lung tissue. Moreover, robustly elevated expression of p16, SASP chemokines (Ccl2, Cxcl10, and Ccl17) and SASP matrix metalloproteinases (Mmp2, Mmp9, and Mmp12) is observed in the macrophages of irradiated lung, which might stimulate a fibrotic phenotype in pulmonary fibroblasts. In summary, irradiation can induce macrophage senescence, and increase the secretion of SASP in senescent macrophages. Our findings provide important evidence that senescent macrophages might be the target for prevention and treatment of RIPF.

## Introduction

The incidence of thoracic cancers has remained generally high over the past few decades, including pulmonary cancer, breast cancer, esophageal cancer, and mediastinal tumors^1^. Thoracic cancer patients received radiation therapy are at risk of developing radiation-induced pulmonary fibrosis (RIPF), which is a common complication of radiotherapy and a major limiting factor for oncotherapy^2–4^. The characters of RIPF include progressive dyspnea, destruction of pulmonary tissues, accumulation of interstitial fluids, and ultimately leading to respiratory failure^4^. The etiology of RIPF remains unclear and effective treatment is lacking in clinic, leading to an extremely poor prognosis in patient with RIPF^4,5^. Therefore, it is urgent to understand the mechanism associated with RIPF and develop novel treatment strategies for prevention and treatment of RIPF.

The culmination of prior studies suggest senescent cells may be crucial contributors to initiation and progression of pulmonary fibrosis (PF), including senescent fibroblast^6–8^ and senescent type II airway epithelial cell^9–11^. Importantly, drugs that can selectively kill senescent cells have been shown to reverse PF and improve pulmonary function^7,11^. Cellular senescence refers to the specific phenomenon wherein previously proliferation-competent cells undergo durable cell cycle arrest in response to various cellular stresses. In addition to growth arrest, senescence is characterized by increased activity of cyclin-dependent kinase inhibitors (p16^INK4a^ and/or p53-p21^Cip1/Waf1^) and senescence-associated β-galactosidase (SA-β-gal), as well as increased secretion of senescence-associated secretory phenotype (SASP), a broad repertoire of cytokines, chemokines, matrix metalloproteinases (Mmps) and growth factors^12–14^. Furthermore, resistance to apoptosis has been described in cellular senescence, which has a stable expression of anti-apoptotic proteins, such as Bcl-2, Bcl-W, and Bcl-XL mediating by changes in p53 pathway^15,16^.

Macrophages are one of the most abundant immune cells in lung tissues (~70% of the immune cells), which are one of the most active secretory cell types in lung tissue and can release a mass of mediators. Macrophage has been recognized to play a vital role in the pathogenesis of PF^17,18^. Depending on local microenvironments, macrophages can be polarized to either classically activated (M1) macrophages or alternatively activated (M2) macrophages. In general, M1 macrophages are responsible for wound healing and can release proinflammatory cytokines and chemokines, such as interleukin-1 (IL-1), IL-6, IL-12, CCL2, CXCL10, and tumor necrosis factor-α (TNF-α), while M2 macrophages are designated to resolve wound healing processes, terminate inflammatory responses and promote collagen synthesis by secreting transforming growth factor-β (TGF-β), IL-10, CCL17, CCL18 and arginase type 1(Arg-1)^18–21^. During irradiation, dysregulation of macrophage activation can contribute to inflammation and also may be associated with excess production of extracellular matrix components and tissue remodeling, which eventually lead to alveolitis and RIPF^22^. However, the role of macrophages in RIPF need to be further explored.

In prior work, we have found ABT-263 selectively kills senescent type II airway epithelial cells and reverses RIPF.^11^ Interestingly, macrophages also exhibited features of senescence with higher expression of SA-β-Gal in irradiated pulmonary tissues in prior study (unpublished data). While senescence primarily refers to replication-competent cells, recent studies have shown that largely postmitotic cell types can also exhibit a senescence program, such as neuron^23^, chondrocyte^24^ and macrophage.^25,26^ Therefore, we hypothesize that senescent macrophages induced by ionizing radiation (IR) play an important role in the development of RIPF.

Experimentation described herein explored the role of senescent macrophages in RIPF pathology during the development of fibrosis. We began by assessing biomarkers of senescence in bone marrow-derived monocytes/macrophages (BMMs) and demonstrated that IR induced BMMs senescence, with increasing expression of SA-β-gal, senescence-associated genes and SASP. Next, using radiation-induced lung injury as a RIPF model, we showed that, similar to senescent BMMs, murine pulmonary fibrosis was characterized by accumulation p16- and SA-β-gal-positive macrophages, with increasing expression of SASP especially chemokines and Mmps. In general, we first found IR could induce senescence of pulmonary macrophage. Second, SASP secreted by senescent macrophages might maintain chronic inflammation microenvironment in lung tissue and ultimately promote the development of fibrosis. Our findings provided important evidence for senescent macrophages as the target for the treatment of human RIPF.

## Results

### Ionizing radiation induced the senescence of BMMs in a time- and dose-dependent manner

**Fig. 1:**
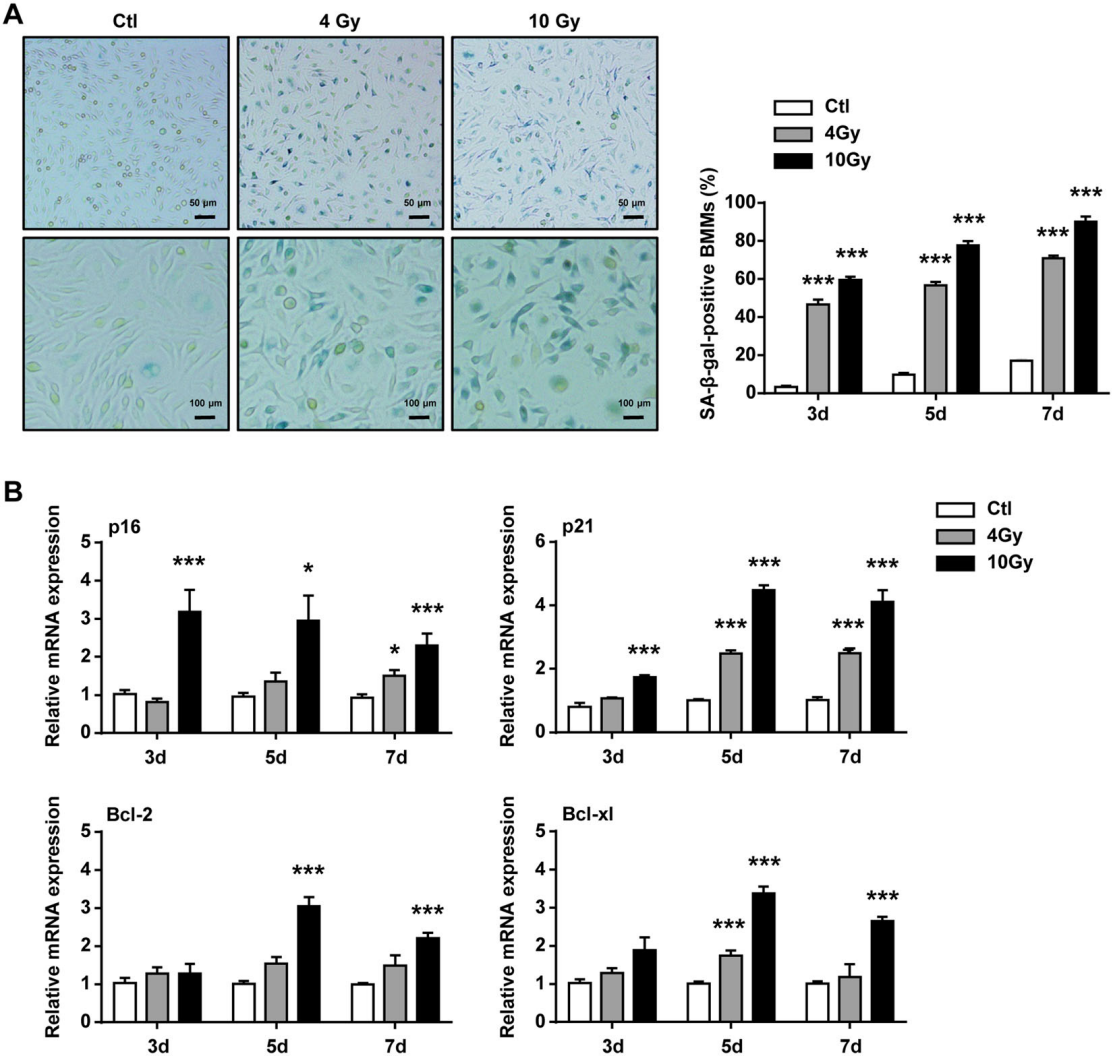
Bone marrow-derived monocytes/macrophages (BMMs) had senescent phenotype after IR. BMMs were exposed to IR at doses of 0 (ctl), 4, or 10 Gy. BMMs were collected at intervals after IR and in controls. **A** Representative images of SA-β-gal staining (scale bar, 100 μm) and quantification of SA-β-gal^+^ BMMs. **B** qRT–PCR expression of p16, p21, Bcl-2, and Bcl-xl were measured relative to Gapdh levels. Data are expressed as mean ± SEM of five independent experiments, ^***^P < 0.001, ^**^P < 0.01 and ^*^P < 0.05 compared with the corresponding controls.

To verify that whether IR could induce macrophage senescence, BMMs were examined at different time after the cells exposed to 4 or 10 Gy irradiation. First, the activity of SA-β-Gal in BMMs was investigated. The ratio of SA-β-Gal^+^ BMMs was gradually increased at 3, 5, and 7 days after irradiation exposure. Whereas 10 Gy irradiation caused a marked increase in the number of SA-β-Gal^+^ cells, when compared with 4 Gy irradiation (Fig. 1A). Then, the expression of senescence-associated genes (p16, p21, Bcl-2, and Bcl-xl) in BMMs were detected and results showed that those genes gradually upregulated in a time- and dose-dependent manner. In addition, senescence-associated genes showed higher expression in 10 Gy irradiation group compared with those in 4 Gy irradiation group (Fig. 1B). These results suggested that macrophages could be induced senescent by 10 Gy irradiation.

### The secretome of senescent BMMs was profibrotic and proinflammatory

**Fig. 2:**
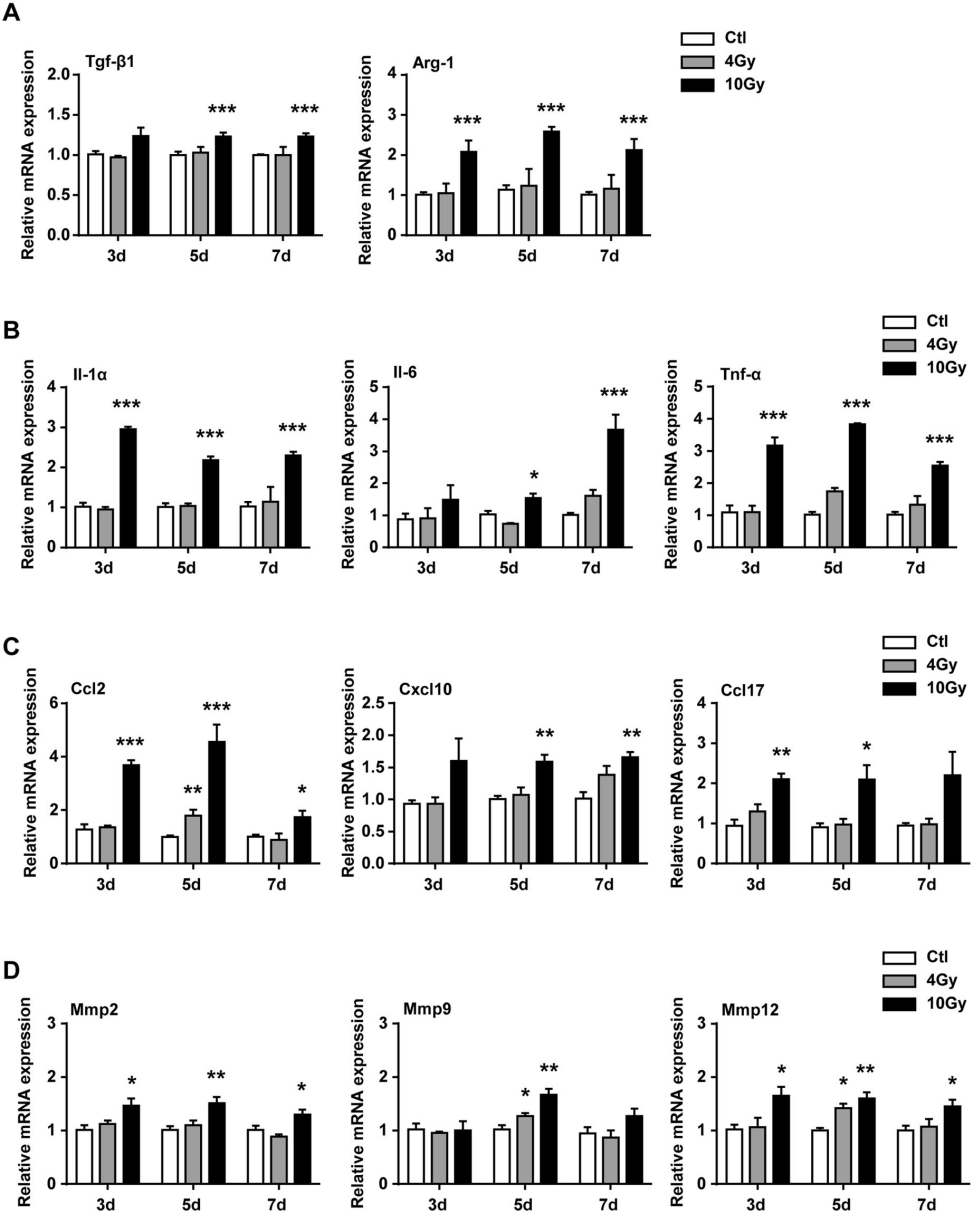
Elevated expression of fibrotic genes and SASP genes in senescent BMMs. BMMs were exposed to IR at doses of 0 (ctl), 4, or 10 Gy. BMMs were collected at intervals after IR and in controls. The expression of **A** fibrotic genes (Tgf-β1 and Arg-1), **B** SASP proinflammatory factors genes (Il-1α, Il-6, and Tnf-α), **C** SASP chemokines genes (Ccl2, Cxcl10, and Ccl17), **D** SASP Mmps genes (Mmp2, Mmp9, and Mmp12) were quantified by qRT–PCR and were expressed relative to Gapdh levels in BMMs. Data are expressed as mean ± SEM of five independent experiments, ^***^P < 0.001, ^**^P < 0.01, and ^*^P < 0.05 compared with the corresponding controls.

Previous studies have reported that senescent cells mediate pulmonary fibrosis via their secretome^27–30^, the dynamic changes of SASP components were detected after BMMs were exposed to 4 or 10 Gy irradiation, including growth and matrix remodeling factors. First, the messenger RNA (mRNA) expression of TGF-β1 and Arg-1, which were important for collagen deposition in RIPF, were robustly upregulated in BMMs at 3 days after 10 Gy irradiation (Fig. 2A). Second, the SASP proinflammatory factors, including Il-1α, Il-6, and Tnf-α, were significantly upregulated in the senescent BMMs (Fig. 2B). Third, the levels of SASP chemokines, such as Ccl2, Cxcl10, and Ccl17, were persistently upregulated in BMMs after IR (Fig. 2C). Forth, the metalloproteinases (Mmps) (including Mmp2, Mmp9, and Mmp12) of SASP matrix in BMMs were also increased after BMMs exposured to 10 Gy irradiation (Fig. 2D). Interestingly, SASP proinflammatory factors, SASP chemokines, and SASP matrix metalloproteinases mRNA expression showed no obvious changes after low doses of irradiation (4 Gy) (Fig. 2A–D).

**Fig. 3:**
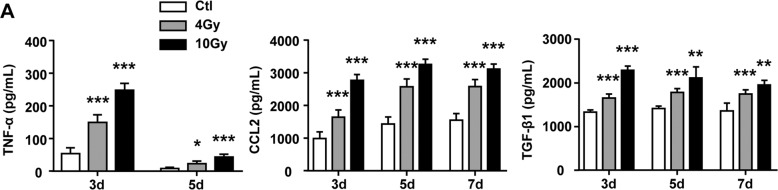
Elevated expression of SASP components in supernatant of senescent BMMs. BMMs were exposed to IR at doses of 0 (ctl), 4, or 10 Gy. The supernatant of BMMs were collected at intervals after IR and in controls. **A** The protein levels of SASP components, including TNF-α, CCL2, and TGF-β1 in supernatant of BMMs. Data are expressed as mean ± SEM of five independent experiments, ^***^P < 0.001, ^**^P < 0.01, and ^*^P < 0.05 compared with the corresponding controls.

Next, the secretion of SASP proinflammatory factors were investigated. Enzyme-linked immunosorbent assay (ELISA) assay results showed that senescent BMMs secreted significant amounts of TNF-α, CCL2, and TGF-β1 in response to IR in dose-dependent fashion (Fig. 3A). Taken together, our results supported that irradiation could increase the secretion of SASP.

### Thoracic IR promoted the SASP in senescent macrophages

**Fig. 4:**
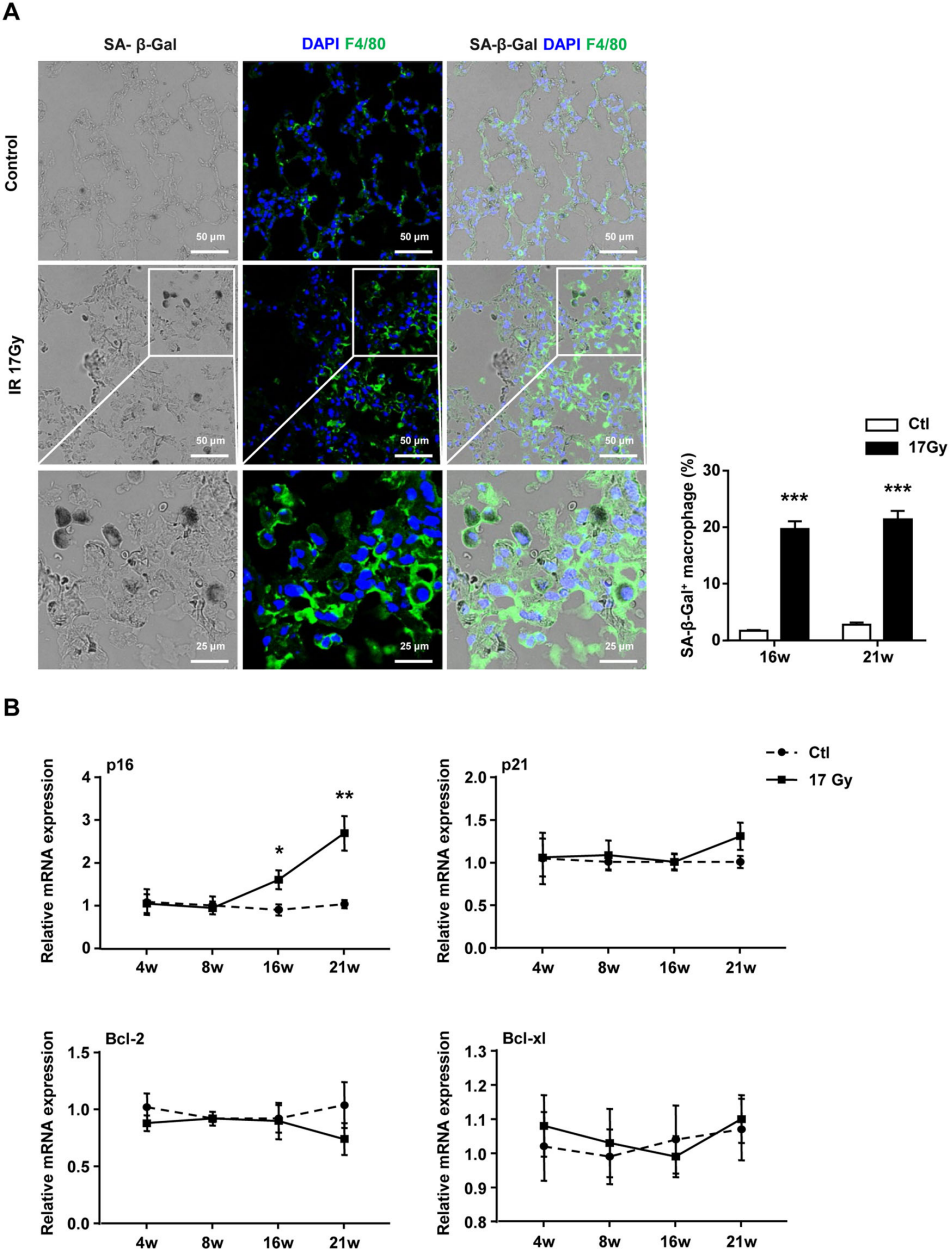
Pulmonary tissue macrophages had a senescent phenotype after thoracic IR. C57BL/6J mice were exposed to a single dose of 17 Gy IR on the right side of the thorax. Samples of right lung tissue were collected at intervals after IR and in controls. **A** Representative images of SA-β-gal staining (scale bar, 25 or 50 μm) and quantification of SA-β-gal^+^ macrophages in right lung tissue. **B** qRT–PCR expression of p16, p21, Bcl-2, and Bcl-xl were measured relative to Gapdh levels in sorted populations of macrophages in right lung tissue. Data are expressed as mean ± SEM of six independent experiments, ^***^P < 0.001, ^**^P < 0.01, and ^*^P < 0.05 compared with the corresponding controls.

While previous studies have provided evidence that exposure of mice to IR can induce a significant increase in senescent cells and contribute to pulmonary fibrosis^11^, the role of senescent macrophages in lung fibrogenesis still need to clarify. In this study, we noted that the percentage of SA-β-Gal^+^ F4/80^+^ macrophage was significantly increased at 16 and 21 weeks after IR, supporting the important role of macrophage in senescence-associated PF (Fig. 4A). To determine whether IR could induce macrophage senescence in vivo, the macrophages (CD45^+^ F4/80^+^) of right lung in C57BL/6J mouse were sorted at 4, 8, 16, and 21 weeks after IR. The expression profiling of senescence-associated genes (p16, p21, Bcl-2, and Bcl-xl) in isolated macrophages (CD45^+^ F4/80^+^) were tested by quantitative reverse transcription PCR (qRT–PCR). The results showed that exposure of mice to IR resulted in a significant increase of p16 mRNA expression in the macrophages from irradiated lung tissues, whereas there was no change of p21, Bcl-2, and Bcl-xl mRNA levels (Fig. 4B).

**Fig. 5:**
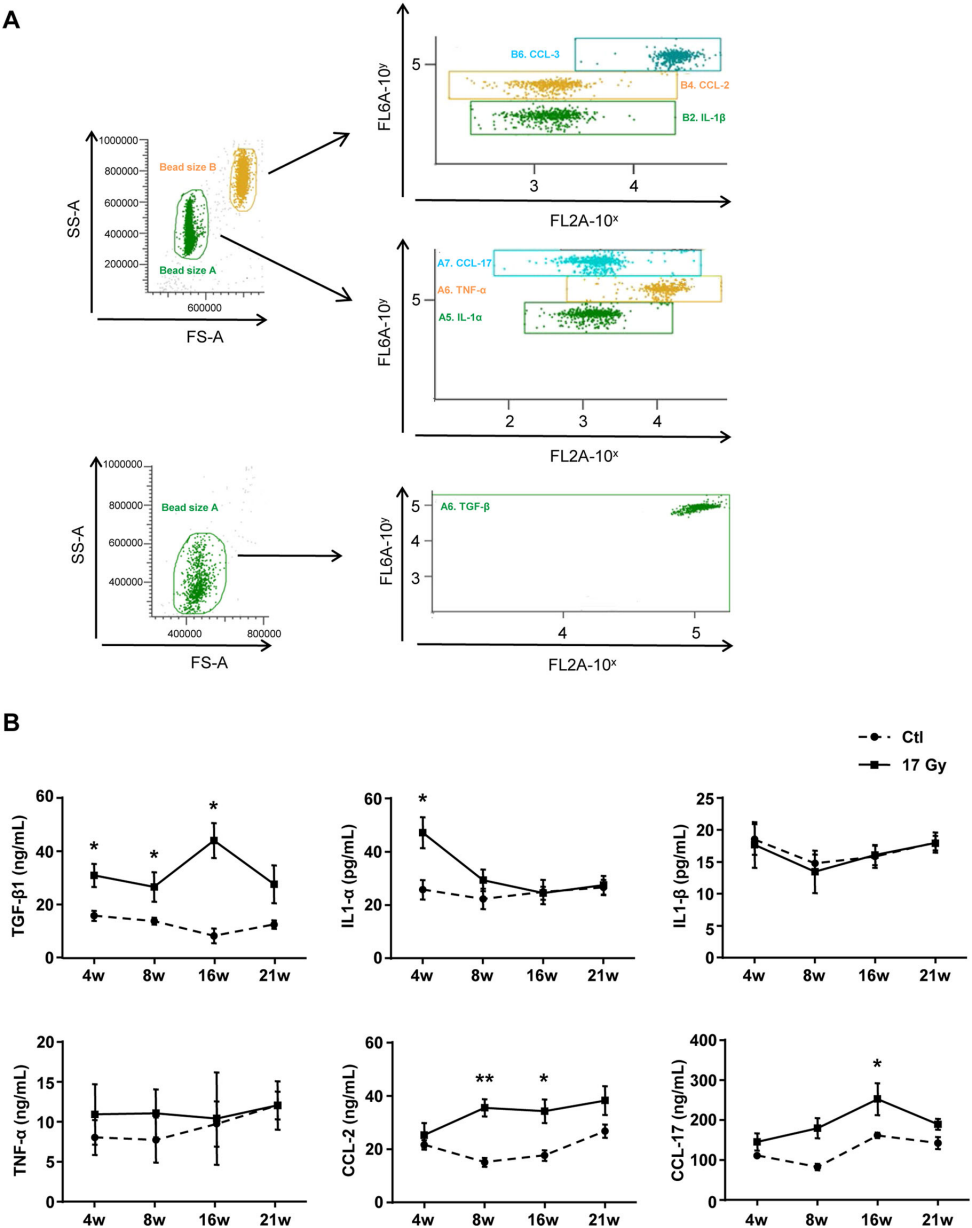
Thoracic IR induced augment of SASP components in lung tissues. C57BL/6J mice were exposed to a single dose of 17 Gy IR on the right side of the thorax. Samples of right lung tissue were collected at intervals after IR and in controls. The lung homogenates were measured by cytometric bead array (CBA) Kit and FCM. **A** Representative images of CBA staining. **B** The protein levels of SASP components, including TGF-β1, Il-1α, Il-1β, TNF-α, CCL2, and CCL17 in homogenates of right lung tissue. Data are expressed as mean ± SEM of six independent experiments, ^**^P < 0.01 and ^*^P < 0.05 compared with the corresponding controls.

Next, how IR stimulated the SASP in lung tissue were investigated. Cytometric bead array (CBA) results revealed that the protein level of TGF-β1 in the irradiated lung tissues was significantly upregulated after IR, which was associated to the development of RIPF (Fig. 5B). Meanwhile, the secretion of IL-1α, CCL2, and CCL17 robustly increased at 4, 8, or 16 weeks after IR, whereas there was no change of IL-1β and TNF-α in the irradiated lung tissues (Fig. 5B). These results suggested that IR promoted the SASP in senescent macrophages, and the secretome of senescent macrophages might robustly stimulate a fibrotic phenotype in the fibroblasts of irradiated lung.

### Thoracic IR elevated the expression of fibrotic genes and SASP genes in senescent macrophages of lungs

**Fig. 6:**
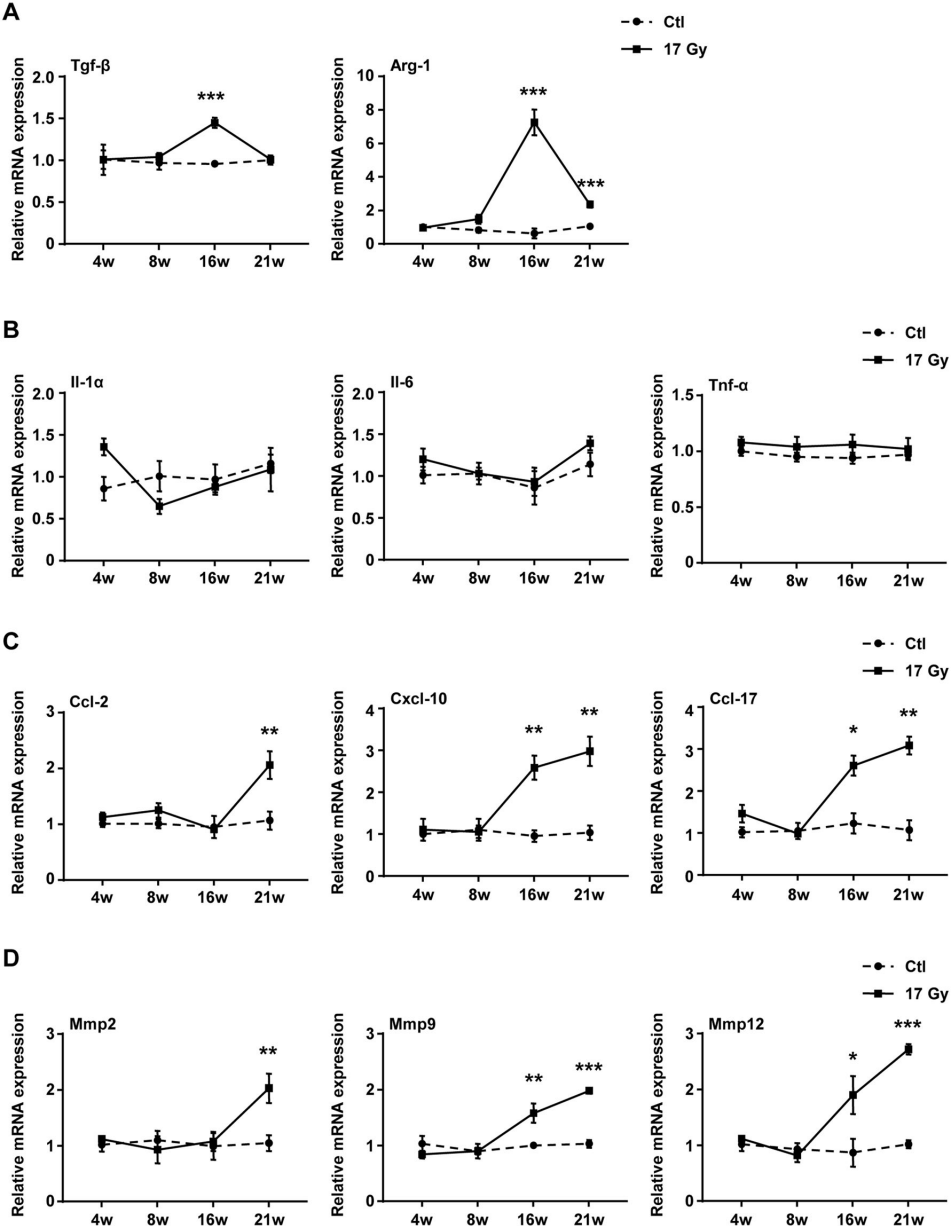
Thoracic IR significantly elevated the expression of fibrotic genes and SASP genes in senescent macrophages of right lung tissue. C57BL/6J mice were exposed to a single dose of 17 Gy IR on the right side of the thorax. Samples of right lung tissue were collected at intervals after IR and in control. The macrophages were sorted by FCM. The expression of **A** fibrotic genes (Tgf-β1 and Arg-1), **B** SASP proinflammatory factors genes (Il-1α, Il-6, and Tnf-α), **C** SASP chemokines genes (Ccl2, Cxcl10, and Ccl17), **D** SASP Mmps genes (Mmp2, Mmp9, and Mmp12) were quantified by qRT–PCR and were expressed relative to Gapdh levels in isolated macrophages. Data are expressed as mean ± SEM of six independent experiments, ***P < 0.001, **P < 0.01, and *P < 0.05 compared with the corresponding control.

To explore the role of senescent macrophage in RIPF, the expression of fibrotic and SASP genes in the macrophages of irradiated lung were further analyzed. qRT–PCR results analysis showed that the mRNA expression of Tgf-β1 in the macrophages from the irradiated lung tissue was significantly elevated at 16 weeks after IR (Fig. 6A). In addition, increased mRNA expression of Arg-1 was also detected in the macrophages from the irradiated lung tissues at 16 and 21 weeks after IR (Fig. 6A).

In parallel, the transcript levels of SASP genes in pulmonary macrophages at different time after irradiation were also monitored. First, qRT–PCR results analysis revealed that the mRNA levels of SASP proinflammatory factors, including Il-1α, Il-6 and Tnf-α remained unchanged in irradiated pulmonary macrophages compared with control group (Fig. 6B). Second, IR treatment significantly increased the mRNA levels of SASP chemokines, such as Ccl2, Cxcl10 and Ccl17 in the macrophages of the irradiated lung (Fig. 6C). Third, the transcript levels of Mmp2, Mmp9 and Mmp12 in irradiated pulmonary macrophages were significantly elevated (Fig. 6D). These data suggested that IR could induce the expression of SASP genes in the macrophages of irradiated lung, indicating that senescent macrophages might be involved in the development of pulmonary fibrosis.

## Discussion

RIPF exhibits a chronic, progressive, irreversible course and is considered refractory to most treatments. There are no FDA-approved pharmacologic agents for the treatment of RIPF. RIPF is characterized by fibroblast proliferation, collagen accumulation and destruction of the normal lung architecture^31^. Cumulatively, it has shown that senescent cells contribute to the development of RIPF, such as senescent fibroblast and type II airway epithelial cell^7–10^. However, the involvement of senescent macrophages remains largely unexplored. In this study, using BMMs and RIPF mice models, for the first time we found that irradiation could induce macrophages senescence and increase the secretion of SASP in vitro and in vivo. Chemokines and Mmps secreted by senescent macrophages might play an important role in the process of RIPF.

Pulmonary macrophages are the predominant effector cells, which play a vital role in PF pathogenesis^18^. Although many mechanisms have been implicated in PF, the pathogenesis of progressive fibrosis is not very clear. A study suggests that cellular senescence can be detected in IPF lung tissue and senescent cells deletion rejuvenates pulmonary function in mice^7^. Cellular senescence follows a DNA damage response increasing of p16, p21, and p53, and subsequent induction of SA-β-gal^32^. Here we demonstrate that the same combination of traits can be acquired by macrophages in vivo and in vitro following IR. Misharin et al.^33^ developed a lineage tracing system that could distinguish alveolar macrophage ontogeny, and they found that monocyte-derived alveolar macrophages persisted in the lung for one year after the resolution of fibrosis by using this lineage tracing system. Moreover, they found that specific genetic deletion of monocyte-derived alveolar macrophages after their recruitment to the lung could ameliorate lung fibrosis, whereas tissue-resident alveolar macrophages did not contribute to fibrosis. Therefore, the BMMs were chosen in our study. In vitro, exposure of BMMs to IR could increase the expression of SA-β-Gal and senescence-specific genes (p16, p21, Bcl-2, and Bcl-xl), suggesting that irradiation could induce BMMs senescence. Recent studies have reported senescent macrophages exhibited some features of senescence, including reduced proliferation, elevated SA-β-Gal activity, increased mRNA expression of SASP, and contributing to the development of aged diseases^9,24,25^. In our RIPF mice model, IR was capable of elevating SA-β-Gal activity of macrophages in irradiated lung tissue from 16 weeks post IR. Furthermore, a substantially increased level of p16 mRNA expression in sorted macrophages at 16 weeks and 21 weeks after IR, suggested that pulmonary macrophages exhibited features of senescence during pulmonary fibrosis instead of radiation pneumonitis in RIPF mice models. These data suggest that IR can simultaneously induce macrophages senescence in vitro and in vivo.

The SASP is one of the hallmarks of cellular senescence, which involves an array of cytokines, chemokines, growth factors, proteases, and profibrotic mediators. The SASP can facilitate wound healing, tissue repair and development through directly influencing the surrounding microenvironment, which may be the mechanism that explains the reason why senescent cells in lung tissue can contribute to PF^34,35^. Furthermore, intervention of SASP from senescent cells improves pulmonary function and physical health^7^. Specifically, the secretome of senescent BMMs contain number of cytokines with established roles in regulating fibrotic and inflammatory aspects of PF, including Tgf-β, Arg-1, Il-1, Il-6, Tnf-α, Ccl2, Cxcl10, Ccl17, Mmp2, Mmp9, and Mmp12. These findings supported the hypothesis that IR could induce BMMs senescence and increase the secretion of SASP in vitro, which showed proinflammatory and fibrotic phenotype. IR could stimulate the secretion of SASP in lung tissue, including TGF-β, CCL2 and CCL17. Combined with elevated SASP mRNA expression (including Ccl2, Cxcl10, Ccl17, Mmp2, Mmp9, and Mmp12) in sorted macrophages after irradiation, we confirmed that senescent macrophages were partly contributed to lung fibrosis. Previous study has verified that elevated SASP mRNA expression (including Il-1α, Tnf-α, Ccl2, Mmp12, and Mmp13) significantly increases in senescent foamy macrophages during atherosclerosis, which has the similar characteristics of SASP in senescent macrophages during lung fibrosis^25^. Thus, we speculated that senescent macrophages could change the lung tissue microenvironment by secreting chemokines and also contributed to lung tissue repair and matrix remodeling by secreting Mmps, which aggravated the occurrence of lung fibrosis.

TGF-β1 is a central fibrosis-associated growth factor, mainly generated by macrophages^36^. During PF, TGF-β1 could induce proliferation and differentiation of fibroblasts, promote synthesis of collagen, and aggregates a variety of inflammatory cells and promotes release of inflammatory factors^37^. Arg-1 in M2 macrophages could contribute to the supply of proline for collagen synthesis, which was very important in the development of pulmonary fibrosis^38^. In line with this, increased expression of Tgf-β1 and Arg-1 has been found in senescent macrophages in vivo. Hence, this subpopulation of sorted macrophages had the same characteristic as senescent cells to be considered a possible contributor to PF through secreting profibrotic factors, chemokines and Mmps, which perpetuated tissue destruction and repair, leading to irreversible lung fibrosis.

Increased senescence has been described for IPF as well as in mouse models of PF in both type II airway epithelial cells and fibroblasts^6–8,39^. However, the short time course and heterogeneity intrinsic to the bleomycin model may limit observable fibrosis formation and/or resolution. Here, we find 10 Gy irradiation can induce the senescence of BMMs and 17 Gy irradiation can induce the senescence of macrophage in irradiated lung tissue, both accompanying with increased fibrosis-associated factor (Tgf-β1, Arg-1), SASP chemokines (Ccl2, Cxcl10, Ccl17), and Mmps (Mmp2, Mmp9, Mmp12). At the same time, our mice models showed extended fibrosis courses, making it easier to understand the role of macrophages and SASP secretion at different time points during RIPF.

In summary, this study showed that IR could induce macrophages senescence and increase the secretion of SASP, and these senescent macrophages accumulated with fibrosis formation in lung tissue. Previous studies have found that the removal of senescent cells can alleviate PF^7,11,39^. These results indicate that senescent cells and SASP may be the targets for the treatment of PF. Although we sorted the pulmonary macrophages at different time points during RIPF, but we did not sort p16- and SA-β-gal-positive macrophages and detect the SASP expression. Therefore, we cannot conclusively determine the effects of senescent macrophages in later stage of RIPF. Future studies using in vivo models targeting specific senescent macrophages are needed to further delineate the contribution to the development of pulmonary fibrosis. In addition, it will be important to further confirm the role of senescent macrophages in human irradiated lung tissues. Taken together, our findings causally implicate senescent macrophages and SASP in fibrotic pulmonary disease, thereby revealing senescent macrophages elimination and SASP blockade as novel therapeutic approaches for the treatment of RIPF.

## Materials and methods

### Mice

Male C57BL/6J mice were purchased from the Institute of Laboratory Animal Sciences, Chinese Academy of Medical Sciences & Peking Union Medical College. In vivo experiment, mice were used at 8-12 weeks of age, weighting 21.0–24.0 g. In vitro experiment, mice were used at 4 weeks of age, weighting 13.0–14.0 g. All experimental procedures used during the research were approved by the Animal Care and Ethics Committee at the Institute of Radiation Medicine, Chinese Academy of Medical Sciences & Peking Union Medical College. (No 1539).

### Isolation and culture of mouse primary BMMs

Granulocyte-macrophage colony stimulating factor (GM-CSF) is required to induce hematopoietic cell differentiation into macrophages. L929 cells can produce GM-CSF. L929 cells were cultured in Dulbecco’s modified Eagle medium (DMEM) supplemented with 10% fetal bovine serum (FBS) in 5% CO_2_ at 37 °C for 7 days (replacing culture medium at day 4 with fresh complete DMEM). L929 cells were then harvested and implanted with 4.7 × 10^5^/bottle and cultured in the presence of DMEM-Gluta MAX medium. After 7 days, the medium was sterilized by filtrating through a 0.22 μm filter and stored at −20 °C, which used as L929-conditioned medium^40–42^.

C57BL/6J mice aged 4 weeks were sacrificed by cervical dislocation at the time of bone marrow (BM) harvest. BM cells were extracted from the femurs by flushing with culture medium. BM cells were cultured in the presence of culture medium supplemented with 10% FBS and 10% L929-conditioned medium (replacing culture medium at day 3 and 5)^40–42^. After 5 days, mice primary BMMs were exposed to different doses of IR.

### IR of BMMs and mice

BMMs were divided into three groups: (a) control group; (b) 4 Gy group; (c) 10 Gy group. BMMs were irradiated with X-rays at a dose rate of 0.9 Gy/min. Mice were exposed to sham IR as control or a single dose of 17 Gy X-rays of IR on the right side of the thorax (dose rate: 3.12 Gy/min).

### Histochemistry and Immunofluorescence staining

BMMs (20,000) were plated in the 24-well plates (Corning, NY, USA). Right lung tissues were fixed in 4% paraformaldehyde and embedded in Tissue Tek OCT compound (Sakura Finetek USA, Torrance, CA, USA). BMMs and frozen lung sections (thickness of 5 µm) were stained using a β-galactosidase staining kit (Cell Signaling Technology, MA, USA) according to the manufacturer’s instructions.

For co-staining, frozen lung sections were permeabilized in 0.3% Triton X-100 for 15 min and then blocked with 3% BSA for 30 min. After that they were incubated with anti-F4/80 rat monoclonal antibody (1:100, Santa Cruz Biotechnology, sc-52664) followed by secondary antibody conjugated with Alexa fluor 488 (1:500, Thermo Fisher Scientific, A-11006).

### CBA detection

Right lung tissues (20–30 mg) were homogenized and lysed in 120 μL lysis buffer. IL-1α, IL-1β, TNF-α, CCL2, and CCL17 in lung homogenates were measured using Biolgend CBA Custom Mouse 10-plex panel Kit (Biolgend, San Diego, CA, USA) and Flow Cytometry (FCM). TGF-β1 was measured using CBA Mouse/Rat Free Active/Total TGF-β1 Assay (Biolgend, San Diego, CA, USA) and FCM.

### Isolation of pneumonocytes and flow-cytometry cell sorting

After fully anaesthetized control and irradiated mice, the right pulmonary tissues were immediately dissected. The lung tissues were finely shredded by scissors in a 60 mm petri dish in cold TESCA buffer containing collagenase (1.5 mg/mL, Sigma-Aldrich, St. Louis, MO, USA) and DNase I (6 μg/mL, Sigma-Aldrich, St. Louis, MO, USA). The mixture was transferred to 15 mL sterile tubes and incubated at 37 °C for 45 min in a water bath. Enzymatic digestion was inactivated by adding DMEM medium containing 10% fetal bovine serum. Cells were then centrifuged at 400 × *g* at 4 °C for 10 min. After treatment with RBC Lysis Buffer (Gibco, Grand Island, NY, USA), single-cell suspensions were then incubated with anti-CD45-PerCp-Cy5.5 (eBioscience, 45-0451-82) and anti-F4/80-PE (BD Biosciences, 565410) for 30 min at 4 °C for cell sorting. In all, 1 × 10^6^ single-cell suspensions were stained with anti-CD45-PerCp-Cy5.5 (eBioscience, 45-0451-82), anti-F4/80-PE (BD Biosciences, 565410), anti-MHCII-FITC(eBioscience, 11-5321-82), and anti-CD206-APC (eBioscience, 17-2061-82) for 30 min at 4 °C and then were analyzed with a Gallios flow cytometer (Beckman, Brea, California, USA). Data were analyzed using FlowJo software. FCM sorting was conducted using a BD FACS Aria II (BD Biosciences, San Jose, CA, USA). FC -sorted macrophages were collected in 1.5 mL Eppendorf tubes containing TRIzol reagent (Invitrogen, Carlsbad, CA, USA), which were subjected to mRNA extraction, complementary DNA synthesis and qRT–PCR analysis.

### Quantitative reverse transcription PCR (qRT–PCR)

Total RNA was extracted from BMMs and sorted pulmonary macrophages using TRIzol reagent (Invitrogen, Carlsbad, CA, USA) according to the manufacturer’s protocol. Taqman MGB probes for the p16^Ink4a^ (catalog number: Mm00494449_ m1), and the housekeeping gene Gapdh (catalog number: Mm99999915_g1) were obtained from Applied Biosystems (Foster City, CA). Each sample was run in triplicate for analysis. At the end of the PCR cycles, melting curve analysis was performed to validate the specific generation of the expected PCR product. The expression levels of mRNAs were normalized to GAPDH and were calculated using the 2^−ΔΔCt^ method. Primers used for qRT–PCR were summarized in Table 1.

**Table 1:** Primer sequence.

Genes		Sequence
*Arg-1*	Sense	TGTGAATGCGCCACATGA
	Antisense	GCAGCTAGAATGAAGGCG
*Bcl-2*	Sense	CCTCCAATACTCACTCTGTC
	Antisense	CTGATGCTGAAGAAGTCGTC
*Bcl-xl*	Sense	GCGTTCAGTGATCTAACATCC
	Antisense	CCGAAAGAGTTCATTCACTACC
*Ccl2*	Sense	TGAACCTTCATGTCTAGGCT
	Antisense	AATAAATATCACACTGCCCGT
*Ccl17*	Sense	GCCATTCCTATCAGGAAGTTG
	Antisense	CTGGACAGTCAGAAACACG
*Cxcl10*	Sense	TAAGCTATGTGGAGGTGCGA
	Antisense	TAGAACTGACGAGCCTGAG
*Il-1α*	Sense	GACTACAGTTCTGCCATTGAC
	Antisense	GTGAGCCATAGCTTGCAT
*Il-6*	Sense	GATAGTCAATTCCAGAAACCGC
	Antisense	TCAGTCCCAAGAAGGCAAC
*Mmp2*	Sense	GACACCTGCACCACCTTA
	Antisense	AAGGTTGAAGGAAACGAGC
*Mmp9*	Sense	CTCTCTACTGGGCGTTAGG
	Antisense	GCAGATTTACAGGACACGG
*Mmp12*	Sense	ACGTAACATTCAGTCCCTCTA
	Antisense	GGTGACAGAAAGTTGATGGT
*p16*	Sense	CTGGGTGCTCTTTGTGTT
	Antisense	GTGCTTGAGCTGAAGCTATG
*p21*	Sense	GTGGGTGTCAAAGCACTTAG
	Antisense	ACAGTCCAGACCAGGATGTTA
*Tgf-β1*	Sense	CACTGCCCATCGTCTACT
	Antisense	ATCATGTTGGACAACTGCT
*Tnf-α*	Sense	GACCCTCACACTCAGATCA
	Antisense	ACTTGGTGGTTTGCTACG
*Gapdh*	Sense	TCATCCCAGAGCTGAACG
	Antisense	TCATACTTGGCAGGTTTCTCC

### Enzyme-linked immunosorbent assay (ELISA) of cytokines

Culture supernatants were stored at −80 °C until the ELISA was performed. The TNF-α, CCL2, and TGF-β1 levels were detected by ELISA kits (DAKEWE Medical Technologies, Shenzhen, China) according to the manufacturer’s instructions. Detectable limits of TNF-α, CCL2 and TGF-β1 were 8 pg/mL, 20 pg/mL, 5 pg/mL, respectively.

### Statistical analysis

SPSS 19.0 software was used to perform the statistical analyses. Data that were normal with homogenous variance were compared by an unpaired Student’s *t*-test, which was used for all comparisons between two groups. Among multiple groups, data were analyzed with one-way analysis of variance with Tukey’s post-hoc comparison when the data were normally distributed. All data were expressed as mean ± standard error of the mean (SEM), *n* ≥ 5. Differences were considered significant at *p* < 0.05. Sample sizes of all experiments were predetermined by calculations derived from our experience. Data were representatives of five times replicates for the in vitro studies. Data were representatives of six times replicates for the in vivo studies. No sample was excluded from the analyses. Animals were randomly assigned during collection, and the strain, sex, and age of the mice were the same, and the data analysis was single masked. Investigators were not blinded to the group allocation during the experiment and outcome assessment.

## Supplementary information

Supplementary Figure Legends

Supplementary Figure. 1

## Data Availability

Data are available upon reasonable request to the corresponding author.
