# Supplementary material for: Potential role of senescent macrophages in radiation-induced pulmonary fibrosis
Source: Cell Death Dis. 2021 May 22;12(6):527. doi: 10.1038/s41419-021-03811-8 (PMC8141056; doi:10.1038/s41419-021-03811-8)
Supplement: Supplementary file 1 — Supplementary Figure Legends [file 41419_2021_3811_MOESM1_ESM.docx]

## Supplementary Fig. 1 The phenotype of macrophages in right lung tissues. C57BL/6J mice were exposed to a single dose of 17 Gy IR on the right side of the thorax. Samples of right lung tissue were collected at intervals after IR and in controls. (A) Gating strategy for isolation of macrophages, M1 macrophages and M2 macrophages. (B) Frequency of macrophages, M1 macrophages and M2 macrophages in right lung tissue. (C) Relative MFI of MHCII on M1 macrophages, MMR on M2 in right lung tissue. Data are expressed as mean ± SEM of six independent experiments, ^***^P<0.001 and ^*^P<0.05 compared with the corresponding controls
