# Supplementary figures and images for: Potential role of senescent macrophages in radiation-induced pulmonary fibrosis
Source: Cell Death Dis. 2021 May 22;12(6):527. doi: 10.1038/s41419-021-03811-8 (PMC8141056; doi:10.1038/s41419-021-03811-8)

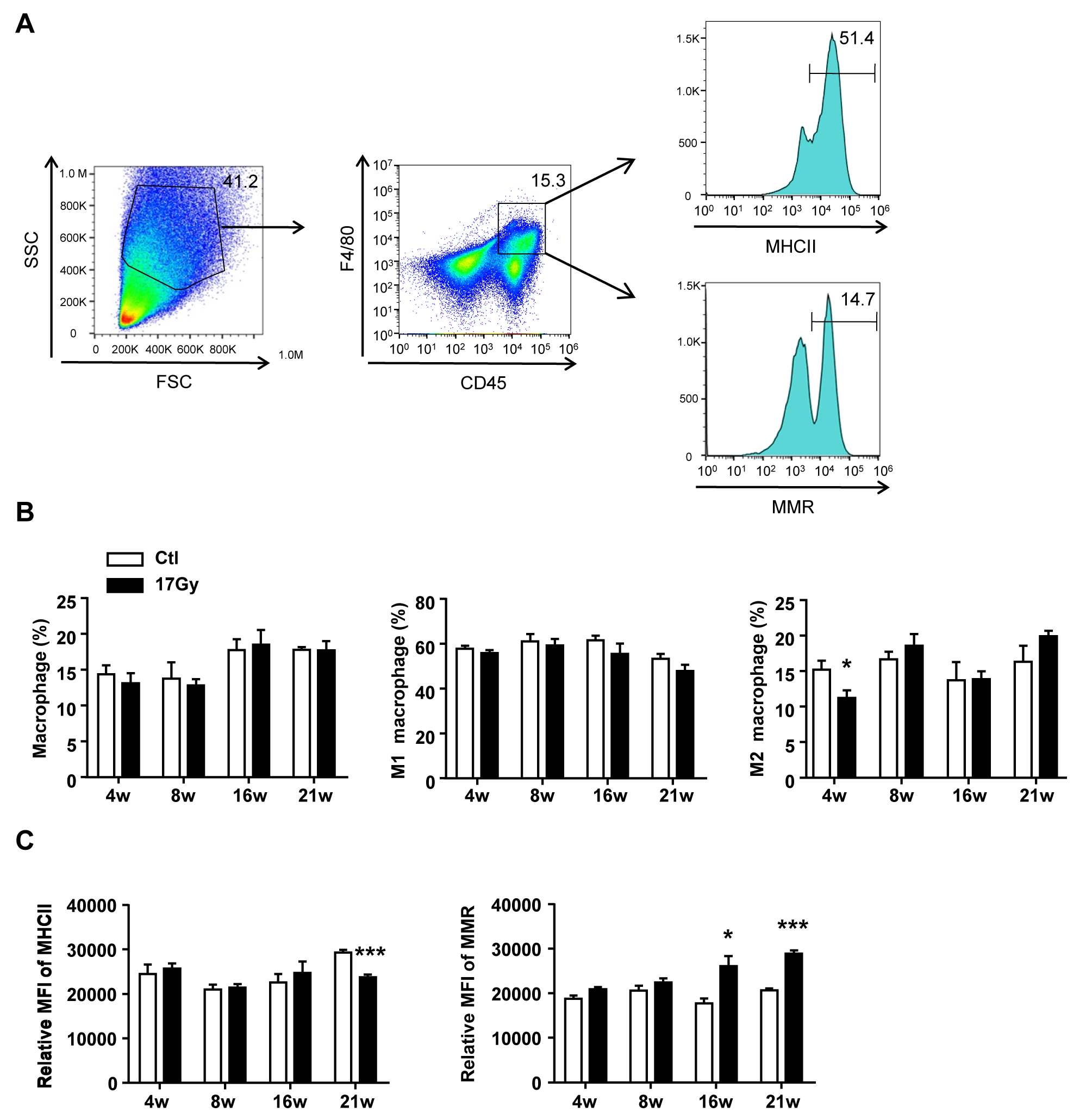

Supplement: Supplementary file 2 — Supplementary Figure. 1 [file 41419_2021_3811_MOESM2_ESM.tif]
